# Physical modeling and validation of porpoises’ directional emission via hybrid metamaterials

**DOI:** 10.1093/nsr/nwz085

**Published:** 2019-07-22

**Authors:** Erqian Dong, Yu Zhang, Zhongchang Song, Tianye Zhang, Chen Cai, Nicholas X Fang

**Affiliations:** 1 Key Laboratory of Underwater Acoustic Communication and Marine Information Technology of the Ministry of Education, College of Ocean and Earth Sciences, Xiamen University, Xiamen 361005, China; 2 Department of Mechanical Engineering, Massachusetts Institute of Technology, Cambridge, MA 02139, USA; 3 State Key Laboratory of Marine Environmental Science, Xiamen University, Xiamen 361005, China; 4 Biology Department, Woods Hole Oceanographic Institution, Woods Hole, MA 02543, USA; 5 Brookline High School, Brookline, MA 02445, USA; 6 Wuhan Second Ship Design and Research Institute, Wuhan 430064, China

**Keywords:** porpoise's physical model, metamaterials, biosonar, directional emission

## Abstract

In wave physics and engineering, directional emission sets a fundamental limitation on conventional simple sources as their sizes should be sufficiently larger than their wavelength. Artificial metamaterial and animal biosonar both show potential in overcoming this limitation. Existing metamaterials arranged in periodic microstructures face great challenges in realizing complex and multiphase biosonar structures. Here, we proposed a physical directional emission model to bridge the gap between porpoises’ biosonar and artificial metamaterial. Inspired by the anatomical and physical properties of the porpoise's biosonar transmission system, we fabricated a hybrid metamaterial system composed of multiple composite structures. We validated that the hybrid metamaterial significantly increased directivity and main lobe energy over a broad bandwidth both numerically and experimentally. The device displayed efficiency in detecting underwater target and suppressing false target jamming. The metamaterial-based physical model may be helpful to achieve the physical mechanisms of porpoise biosonar detection and has diverse applications in underwater acoustic sensing, ultrasound scanning, and medical ultrasonography.

## INTRODUCTION

Directional emitters can transmit waves to form a narrow beam, increasing the detection resolution and energy in the direction of interest. They have been widely used in applications, such as wireless communications, weather radars, quantum emitters, underwater sonars, medical ultrasonography, etc [1,2]. Existing man-made transducers with simple structures have to conquer the size–wavelength limitation, i.e. the size of a directional transducer should be sufficiently larger than the wavelength, to realize directivity [3]. Natural biosonar systems in animals have evolved for millions of years to suit highly variable environments [4–6]. Echolocating bats and porpoises emit sounds, forming directional beams that forage in noisy environments. Our recent study has shown that porpoises have evolved a multiphase forehead complex for underwater acoustic manipulation [5]. However, biosonar systems in porpoises, which are protected animals, have limited access and are less programmable. Therefore, it would be valuable to design better artificial structures starting with that naturally obtained to improve man-made acoustic devices, which is an open possibility by applying evolutionary algorithms.

Metamaterials provide a promising tool to design physical properties through the programmable design of microstructures [7–15]. Acoustic metamaterial can achieve a variety of remarkable acoustic manipulations, such as directional emission [10], negative refraction [14], negative modulus [11], invisible cloaking [8,12], and extraordinary transmissions [9]. A conceptual metamaterial model has been proposed to approach the gradient index property of the Yangtze finless porpoise's melon. This model, however, is over-simplified and could not represent complex biological morphology and variant tissue properties [3]. There is a high demand for using complex metamaterial to mimic the complex physics of animal biosonar for underwater acoustic detection.

Here, we propose a physics-based porpoise model (PPM) via hybrid metamaterial. Based on the complex anatomical and acoustical properties of the porpoise's head, we composed a PPM with steel maxilla, air structures, steel–water and aluminum–water composites. A physical porpoise model with programmable microstructure has been used for the first time to reconstruct complex biosonar components. In comparison with omni-directional systems, the PPM device numerically and experimentally increased directivity and main lobe energy over a broad bandwidth. Its application in underwater target detection is also investigated.

## STRUCTURE DESIGN AND ASSEMBLY OF THE PPM

**Figure 1.**
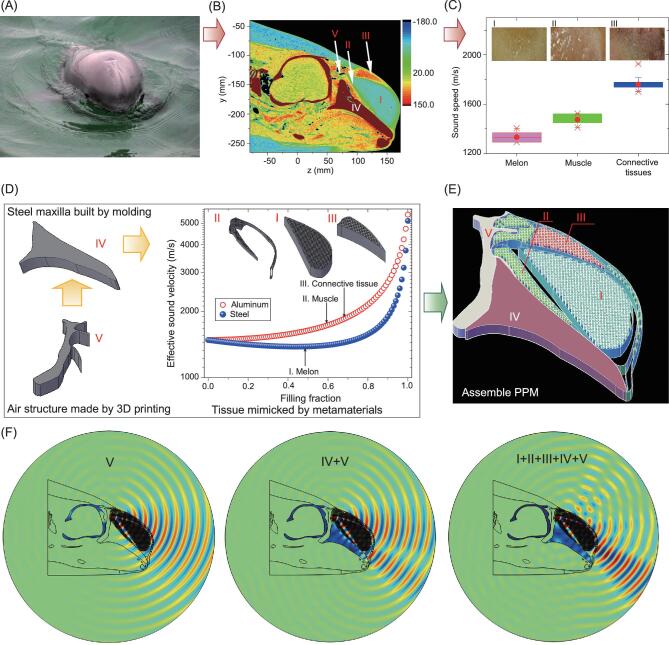
Design of PPM device. (A) Image of a finless porpoise. (B) Computed tomography imaging of the porpoise, where I, II, III, IV, and V correspond to melon, muscle, connective tissue, skull, and air sacs, respectively. (C) Sound velocity distributions of melon, muscle, and connective tissues. (D) Schematic illustration of the fabrications of air structure, steel maxilla, and hybrid metamaterials. Melon, muscle, and connective tissue were mimicked by using a steel cylinder array with *φ* = 0.44, an aluminum cylinder array with *φ* = 0.62, and a steel cylinder array with *φ* = 0.66, respectively. (E) Assembly of the PPM device. (F) Acoustic beam formations of a model with air component V, a model with V and solid structure IV, and the PPM model with I, II, III, IV, and V, where frequency 30 kHz was used.

Porpoises develop highly sophisticated biosonar for spatial orientation and food acquisition (Fig. 1A). According to computed tomography imaging and tissue experiment in our recent studies [5,16,17], the porpoise's forehead includes complex acoustic structures such as soft tissues (melon I, muscle II, connective tissue III), skull IV, and air sacs V (Fig. 1B). Sound speed significantly increases from the inner forehead (melon) to the outer layers (muscle and connective tissue), as shown in Fig. 1C. The biology study by imaging the porpoise's forehead provides information on the acoustic structure and parameters for physical modeling. Inspired by these complex multiphase properties of the porpoise's forehead, we designed a PPM detector in Fig. 1D according to the following procedures: (1) A solid maxilla was fabricated by molding a steel plate with a curved shape; (2) The air structure was made by a 3D printed acrylic mold; (3) Hybrid metamaterials were composed to achieve effective sound speeds of the porpoise tissues according to the effective medium theory [18–21] as:

$$\begin{equation*}
{c_{{\rm{eff}}}} = \sqrt {\frac{{{B_{\rm{w}}}{B_{\rm{a}}} \times [({\rho _{\rm{a}}} + {\rho _{\rm{w}}}) - ({\rho _{\rm{a}}} - {\rho _{\rm{w}}})\phi ]}}{{{\rho _{\rm{w}}} \times [({\rho _{\rm{a}}} + {\rho _{\rm{w}}}) + ({\rho _{\rm{a}}} - {\rho _{\rm{w}}})\phi ] \times [{B_{\rm{w}}} + ({B_{\rm{a}}} - {B_{\rm{w}}})\phi ]}}} ,(1)
\end{equation*}$$

where $\phi $ is the filling fraction of the metal cylinder, ${\rho _{\rm{w}}}$ and ${\rho _{\rm{a}}}$ are the densities of water and metal, and ${B_w}$ and ${B_a}$ are the bulk moduli of water and metal, respectively. Square arrays of metal cylinders with a lattice constant of 4 mm were used to make the composites. The steel–water composite with *φ* = 0.44, aluminum–water composite with *φ* = 0.62, and aluminum–water composite with *φ* = 0.66 mimicked the melon, muscle, and connective tissue, respectively. The sound speeds of the system are tunable by changing the filling fraction of these metamaterials. Clearly, the effective medium theory provides a programmable way to design PPM by parameterizing the sound speeds in the porpoise (Fig. 1D). We then assembled composites to form a physical model to approach the complex morphology and sound speed distribution of the porpoise (Fig. 1E).

We first tested how these hybrid structures in PPM worked together to manipulate sounds into directional beams using full wave simulations (Fig. 1F). The solutions of wave equations with boundary conditions were numerically derived by using COMSOL Multiphysics modeling software (Stockholm, Sweden) [5]. The acoustic pressure and normal velocity at the boundaries of fluid media are continuous, while normal velocity and mechanical stress at fluid–solid boundaries are also continuous. In air structure, water, and soft tissues, longitudinal waves were obtained, while in skull and target, both shear and compressional waves were obtained. For the frequency 30 kHz, the lattice constant 4 mm of these cylinders was much smaller than wavelength, suggesting that the hybrid part mimicked tissues as metamaterial. For the model with the air component V alone, the left air cavity acts as a curved boundary to reflect the acoustic wave forward. For the model with air and solid components, the skull reflects waves at the rostrum–melon interface, and meanwhile induces interfacial waves within the solid maxilla. Scholte waves appear at the solid–water interface. Together with these two components, metamaterials with the programmable parameter $\phi $ in PPM further function as an inhomogeneous waveguide to bend the waves. Metamaterials alone cannot focus acoustic waves. Actually, the PMM includes an air component, hybrid metamaterials, and the skull. These multiphase materials are the key to producing a directional acoustic field.

## NUMERICAL SIMULATIONS OF BIOSONAR AND PHYSICAL MODELS

**Figure 2.**
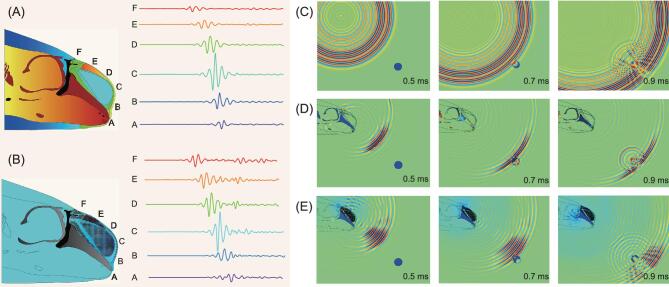
PPM capable of propagating directional waves. (A) Pressure distribution at six positions of the porpoise's biosonar surface. (B) The corresponding distribution of the PPM device. (C) Acoustic wave propagation of the omni-directional system without PPM, where a short pulse at a central frequency of 30 kHz was used; the snapshots correspond to the propagation times at 0.5 ms, 0.7 ms, 0.9 ms, respectively. (D) Propagation plots of the biosonar model, where the main beam angle and −3 dB beam width were estimated as 52° and 24°, respectively. (E) Propagation plots of the PPM device, where the main beam angle and −3 dB beam width were estimated as 58° and 18°, respectively.

We further investigated the performance of the PPM system on producing a transient directional wave to detect a target. For a point source with short duration signal, acoustic pressure non-uniformly distributes at A, B, C, D, E, and F in both the porpoise's biosonar and PPM (Fig. 2A and B). Acoustic pressure had a maximal amplitude at D but significantly decreased at other positions. It differed from the uniform pressure distribution produced by the omni-directional point source. The performance of the omni-directional sound source, a porpoise's biosonar, and PPM in detecting an underwater aluminum target with a diameter of 10 cm is compared in Fig. 2C–E, where the snapshots correspond to the propagation times at 0.5 ms, 0.7 ms, and 0.9 ms, respectively. For the point source without PPM, acoustic pressure was evenly distributed omni-directionally, which made it difficult to separate the scattered wave from the background due to interference. In comparison, PPM modulated the acoustic waves into a main beam, which resulted in the scattered waves from the target being readily distinguishable in the background field. The main beam angle and −3 dB beam width of the PPM device were estimated as 58° and 18°, which were close to the values of 52° and 24° of the porpoise's biosonar at the central frequency of 30 kHz. The PPM device effectively reconstructed the natural porpoise biosonar in beam directivity [5] and can be applicable in target detection. Note that the impulse duration of PPM was longer than that of the porpoise's biosonar, suggesting that it has a lower spatial resolution. It may be associated with multiple scatterings in hybrid metamaterials. Further studies should be performed on physical models to improve the spatial resolution for target detection.

## EXPERIMENTAL VALIDATION OF THE PHYSICAL MODEL

**Figure 3.**
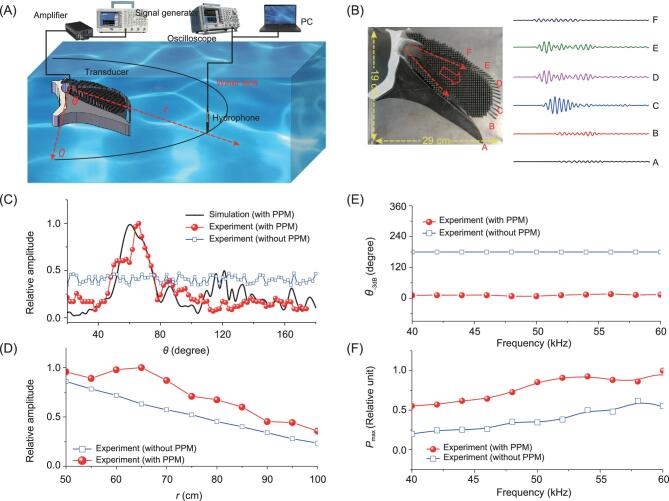
Experimental validation of the directional PPM system. (A) Systematic diagram of the experimental setup. (B) Measured pressure distribution at positions A, B, C, D, E, and F of the PPM surface. (C) Experimentally measured and numerically simulated pressure distributions with respect to angle *θ* of the device with and without PPM. (D) Measured main lobe amplitude with respect to distance *r* of the device with and without PPM. (E) Frequency responses of the beam widths of the devices. (F) Frequency responses of the main lobe amplitudes of the devices.

To validate the directional property of the PPM device, we performed acoustic transmission experiments in an anechoic water tank (Fig. 3A). The tank, which has dimensions of 1.5 m × 1.5 m × 1 m, was covered by sound-absorbing material to emulate the condition of a free acoustic field as much as possible. An omni-directional underwater acoustic transducer with a diameter of 3 cm and a flat frequency response from 40 to 60 kHz was used to transmit a tone-burst signal with five-cycle duration. The PPM in experiments was a 3D device with thickness 5 cm, length about 29 cm, and height about 19 cm. The above simulations used the center frequency at 30 kHz to reveal the principle of PPM, while the following experiments used the frequency range from 40 to 60 kHz in order to ensure that the PPM had the ability of broadband beam control. A power amplifier (ATA-4011, Aigtek) was applied to improve the signal-to-noise ratio. The signals were recorded by a broadband hydrophone (8103, B&K, Denmark). This signal was then A/D converted with a sampling rate of 400 kHz and measured 10 times to reduce noise perturbation and test reproducibility. Figure 3B shows the measured pressure distributions of A, B, C, D, E, F at the PPM surface for a frequency of 60 kHz. The acoustic pressure had a maximum value at position C, which rapidly decreased at other positions. The measured main beam angle and −3 dB angle were 62° and 10°, which are comparable with the simulated results (Fig. 3C). In comparison with the omni-directional transducer, the PPM emitter has a great advantage in improving angular resolution. Numerical simulations showed good consistency with experimental measurements. In addition, Fig. 3D gives the measured result about the maximum amplitude of the main lobe with respect to distance *r.* Figure 3E and F shows the frequency responses of the half-power beam width, and the frequency responses of the main lobe amplitude of the device with and without PPM. The main lobe energies of the PPM device are significantly higher (about 6.5 dB) than those without PPM. For the frequency range from 40 to 60 kHz, −3 dB bandwidths with PPM were significantly lower than those without PPM. The experimental results demonstrate that PPM is a broadband control device that has a directional capability in a continuous frequency range.

## APPLICATION OF THE PPM DEVICE

**Figure 4.**
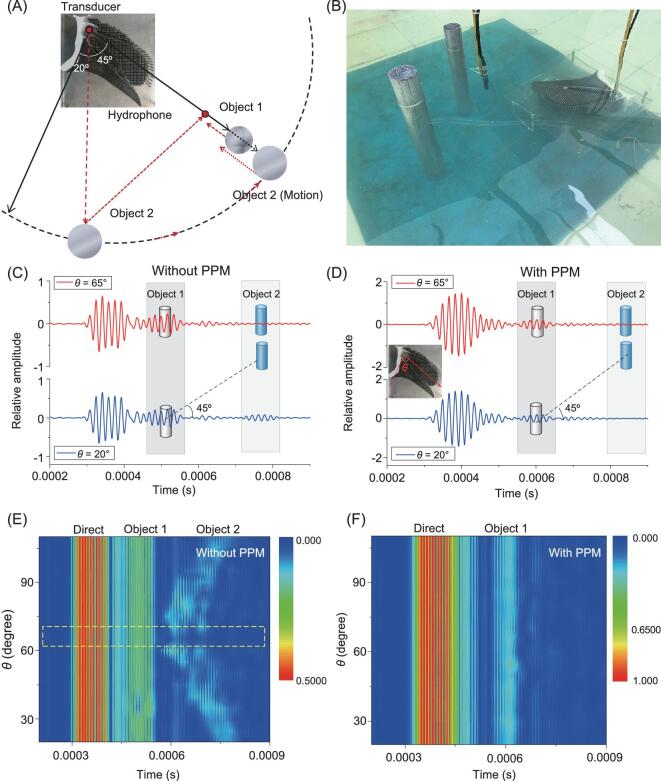
Directional target detection application of the PPM device. (A) Schematic showing the experimental setup. (B) Photograph of the underwater target detection. (C) The measured pressures of the system without PPM at *θ* = 20° (lower) and 65° (upper), where Object 1 and its jamming Object 2 were used for underwater detection. (D) The pressures of the system with PPM at *θ* = 20° (lower) and 65° (upper). (E) Pressure distribution with respect to *θ* of the system without PPM, where the dashed line represents the range that Object 2 is behind Object 1. (F) Pressure distribution with respect to *θ* of the system with PPM.

Finally, we investigated underwater acoustic detection application by using PPM to improve target recognition. The aluminum Object 1 (50 cm length, 5 cm diameter) was placed 1 m away from the source, which was also located along the main beam axis. A second aluminum target, Object 2, (50 cm length, 6 cm diameter) was placed 10 cm behind Object 1 as a jamming object. The hydrophone was placed 10 cm away from the object, between Object 1 and the transducer. In the experiment, the motion of Object 2 was centered on the transducer (Fig. 4A). The sound-absorbing material could receive clean direct and scattered signals from Object 1 and Object 2 (Fig. 4B). The measured pressures of the detector with and without PPM at *θ* = 20° and 65° are compared in Fig. 4C and D, respectively. For aluminum objects under water conditions, there was significant acoustic–solid coupling to produce acoustic scattering. Under excitation from the direct signal without PPM, the scatters vibrated and excited various acoustic modes. The existing modes superposed with each other, and they interfered with the omni-directional direct wave. Therefore, the time difference in the scattered signal was longer than that of the direct signal. However, for PPM, the multiple scattering caused the longer duration time of the direct signal, while the acoustic directivity suppressed the interference between the scattered waves with the direct waves, and thus decreased the duration time of the scattered signal. When *θ* was within the range given by the dashed line in Fig. 4E, the echo from Object 1 can be detected by both systems with and without PPM. However, when *θ* was out of this beam width, the detector without PPM received scattered echoes from both Object 1 and the jamming Object 2, while the detector with PPM received a scattered echo only from Object 1 (Fig. 4F). The directional property of PPM eliminated noises coming from regions located outside of the acoustic view (i.e. the beam width) and reduced the interference of the false target. Thus, the PPM device processes a similar acoustic emission technique to porpoises’ biosonar to improve the energy in the direction of interest, signal-to-noise ratio, and target detection ability, which is of great significance for applications in underwater acoustic detection.

Finless porpoises (*Neophocaena phocaenoides*) inhabiting Indo-Pacific coastal waters produce directional echolocation clicks to track prey [22]. Their capability to detect underwater targets with different materials has been experimentally revealed [23]. During the porpoise's ultrasound emission, the echolocation sound source has been identified as the monkey lips/dorsal bursae complex located below the blowhole in the paired nasal system [5]. The generated sound is reflected by air structures, propagated through the forehead tissues, and transmitted into water. The melon and surrounding tissue function as an adjustable structure, and their shape changes induced by accessory muscles can change the acoustic beam properties, suggesting that the shapes of hybrid metamaterial may be important for beam forming. In addition, a porpoise has a 3D biosonar system. Since the effective medium theory [18–21] is applicable for 3D systems to design gradient sound speed distributions, the hybrid metamaterial design in this study may be capable of 3D beam steering. Our recent computer modeling study suggests that porpoise tissues might be valuable for the future development of man-made metamaterials [5], and that this physical modeling study further provides direct evidence of the application of artificial metamaterials to approach the directional properties of porpoises’ ultrasound emission.

## SUMMARY AND PERSPECTIVE

In this paper, we have proposed a hybrid metamaterial device inspired by porpoise biosonar. In comparison with existing artificial devices [3,7–15,24–26] used in wave manipulation, PPM had a more complex geometry and hybrid composites while showing efficiency in target detection. Its sound speed distribution was parameterized using the effective medium theory. The significant improvement in angular resolution was shown through numerical simulations and experimental measurements. The measured main lobe energy was about 6.5 dB higher than that without PPM over a broad bandwidth. The PPM system detected the target within its beam width, but suppressed false target jamming out of its acoustic view, suggesting a qualitative consistency with the porpoise biosonar in directional detection [5]. Existing metamaterial designs may be challenging in broadband manipulation of subwavelength underwater acoustic waves. The proposed porpoise-inspired design shows that complex metamaterial geometries and multiple-phase composites lead the subwavelength acoustic source to produce directional underwater beams. It provides a valuable bioinspired model for developing man-made metamaterials to control underwater acoustic propagation. Furthermore, this hybrid metamaterial system offers attractive advantages such as programmability, reproducibility, and artificiality, which are considered challenging for animal biosonar systems. The parameterized sound speeds of the artificial structures can be adjusted to manipulate acoustic function as needed. Acoustic metamaterials might be able to break conventional barriers in artificial devices [7,14], while this study further suggests that metamaterial might provide a novel application to achieve animal biosonar properties. Thus, the proposed device combines the features of biosonar and metamaterials, paving a new way to approach powerful bioinspired sonar functions. Its diverse applications for underwater acoustic sensing, medical ultrasonography, and related acoustic applications are promising.
